# Effect of Solid Biological Waste Compost on the Metabolite Profile of *Brassica rapa* ssp. *chinensis*

**DOI:** 10.3389/fpls.2018.00305

**Published:** 2018-03-16

**Authors:** Susanne Neugart, Melanie Wiesner-Reinhold, Katja Frede, Elisabeth Jander, Thomas Homann, Harshadrai M. Rawel, Monika Schreiner, Susanne Baldermann

**Affiliations:** ^1^Leibniz Institute of Vegetable and Ornamental Crops, Großbeeren, Germany; ^2^Department of Food Chemistry, Institute of Nutritional Science, University of Potsdam, Nuthetal, Germany

**Keywords:** metabolite profiling, LC/MS, pak choi, carotenoids, phenolic compounds, glucosinolates

## Abstract

Large quantities of biological waste are generated at various steps within the food production chain and a great utilization potential for this solid biological waste exists apart from the current main usage for the feedstuff sector. It remains unclear how the usage of biological waste as compost modulates plant metabolites. We investigated the effect of biological waste of the processing of coffee, aronia, and hop added to soil on the plant metabolite profile by means of liquid chromatography in pak choi sprouts. Here we demonstrate that the solid biological waste composts induced specific changes in the metabolite profiles and the changes are depending on the type of the organic residues and its concentration in soil. The targeted analysis of selected plant metabolites, associated with health beneficial properties of the Brassicaceae family, revealed increased concentrations of carotenoids (up to 3.2-fold) and decreased amounts of glucosinolates (up to 4.7-fold) as well as phenolic compounds (up to 1.5-fold).

## Introduction

Rising world population, globally changing climate, intensification and competition of biomass usage as source of bioenergy and urban horticultural practice are only a few examples which enhance people's concern about the efficient, multiple use of bio-mass and recycling of residues. Biological waste is generated at various steps within the food production chain, e.g., production of dairy products, oil industry, beverage producers including breweries, processing of fruits and vegetables, and coffee producers (e.g., Mahro et al., 2015). These industries generate large quantities of biological waste and 13 million tons are produced in Germany per annum (Mahro et al., 2015). Much of these wastes are either burnt or treated by oxygen-driven biological methods, such as composting, which serves a dual purpose, i.e., valorization *via* material value as well as decreasing the pollution potential (Murthy and Naidu, 2012; Shemekite et al., 2014). On the other hand biological food waste is a reservoir of complex carbohydrates, proteins, lipids, and nutraceuticals and can form the raw materials for commercially important metabolites. Different modes of pre-treatments, followed by recovery procedures have been applied to attain value-added products (natural antioxidants, vitamins, enzymes, cellulose, starch, lipids, proteins, and pigments) of spent coffee of high significance to pharmaceutical, cosmetic, and food industries (Murthy and Naidu, 2012). The valorization of the solid coffee by-products in the non-food sector is also gaining more importance (Murthy and Naidu, 2012). During the technological processing of fruits and berries, large amounts of solids remain containing pulp, skins, seeds etc. In Germany, industrial processing produces around 7 million tons and in Europe ca. 30 million tons of such wastes. Aronia and hop wastes represent two of these potential wastes. In case of berries, such wastes have also been termed as “pomace.” The major part of these residues is either used for animal feed or is more generally disposed–the disposal being costly. Aronia pomace and spent hops have been utilized to extract bioactive antioxidant phenolics (Baranowski et al., 2009; Yui et al., 2013; D'Alessandro et al., 2014; Brazdauskas et al., 2016; Takahashi and Osada, 2017; Xu et al., 2017). Furthermore, compost can enhance the organic content of soil. In this context, the use of organic soil amendments can influence soil fertility, microbial soil communities as well as plant growth and health. The use of compost is positively associated with higher plant available water holding capacity and lower bulk density and can foster beneficial microorganism populations (Grassi et al., 2015; Pan et al., 2016). Studies report the potential of using diverse cover crop green manure in field vegetable production; however the potential of organic waste as compost arising within the food production chain remains to be investigated. Green manures contribute to nitrogen, phosphorus, and potassium contents of soil (Thönnissen Michel, 1996; Diniz et al., 2007; De Neve, 2017; Verlinden et al., 2017). Moreover, eliminating synthetic fertilizers in agriculture is an issue of global concern, due to the associated decrease in soil fertility after its use, pollution, and contamination of produced horticultural products; all with possible relations to human health. The effect of organic production on the secondary metabolite profiles has been Investigated (Soltoft et al., 2011), however it remains unclear how the usage of residues of the food production chain modulates secondary plant metabolite profiles.

Apart from sustainable production and processing in recent years demands concerning the quality of foods is changing and consumers expect safe, high-quality plant foods. Quality is determined by a variety of aspects, including appearance, aroma, and taste, but increasingly also of value-giving plant metabolites. Secondary plant metabolites are defined as bioactive non-nutrients in fruits, vegetables and other plant foods and have been linked to reducing the risk of chronic diseases, i.e., cardiovascular or aging eye diseases (Hartley et al., 2013; Larsson et al., 2013). Secondary plant metabolites include carotenoids, glucosinolates, and phenolic compounds.

Here we report the effect of biological waste used as compost on the content of secondary plant metabolites in *Brassica rapa* ssp. *chinensis* (pak choi). Pak choi can be cultivated under a variety of agro-climatic conditions and is a fast growing green leafy vegetable, which is very frequently consumed in Asian countries and in steadily rising quantities in Europe. As with other species, the amount of bioactive substances is known to depend on several parameters, including genotype, growing conditions comprising also soil characteristics, and developmental stage.

Pak choi is rich in secondary plant metabolites and contains considerable amounts of β-carotene and lutein with average concentrations of around 4.5 and 8 mg/100 g edible fresh weight (FW), respectively (Reif et al., 2013). The concentration of β-carotene in other vegetables such as broccoli (0.8–2 mg/100 g FW), cauliflower (white, 0.01 mg/100 g FW), or Chinese cabbage (0.4–3 mg/100 g FW) is substantially lower (Harbaum et al., 2007).

As *Brassica* species pak choi is rich in glucosinolates, too. The concentrations of these valuable secondary metabolites are ranging from 100 to 300 mg/100 g FW (Wiesner et al., 2013b). The concentrations are comparable to other vegetables such as cabbage (90–200 mg/100 FW), kohlrabi (109 mg/100 g FW) and more than in cauliflower (60 mg/100 g FW) or broccoli (80 mg/100 g FW) (Sones et al., 1984; Kushad et al., 1999).

Moreover, *Brassica* vegetables are a rich source for phenolic compounds. Diverse phenolic compounds have been described in vegetables of the Brassicaceae family. These include the flavonoids kaempferol, quercetin, and isorhamnetin derivatives (Schmidt et al., 2010). As in other plants these core structures are glycosylated with sugar moieties and can be additionally acylated (Schmidt et al., 2010). Former studies presented a variety of kaempferol, quercetin, and isorhamnetin derivatives as well as various hydroxycinnamic acids in pak choi (Harbaum et al., 2007). The concentration of flavonoid derivatives in the leaf ranged from 4.68 to 16.67 mg/g and the concentration of hydroxycinnamic acid derivatives ranged from 1.48 mg/g of to 5.83 mg/g of dry matter (Rochfort et al., 2006).

We used solid biological waste as compost of the food production of coffee, aronia and hop and were particularly interested if these can modulate the phytochemical concentrations and profiles. We applied LC-ToF-MS to investigate the effect of the organic residues on metabolite profiles in pak choi. In addition, targeted analyses of carotenoids and chlorophylls, glucosinolates, and phenolic compounds were performed.

## Materials and methods

### Plant material

Sprouts of pak choi (*Brassica rapa* ssp. *chinenesis*) cv. Black Behi were grown in green houses in Grossbeeren, Germany in summer 2015. The sprouts were grown on soil and mixtures of soil and compost. The soil used consisted of 35% vulcan clay, 50% turf, and 15% bark humus (Fruhstorfer Erde, Vechta, Germany, for further characterization see Table 1). Five or 10% of the soil was replaced by compost (percent by weight) since these quantities did not impact the plants' development. The biological waste used as compost was dried and grounded to a powder prior mixing with the soil. Spent coffee grounds were obtained from DEK Deutsche Extrakt Kaffee GmbH Berlin (Berlin, Germany) and the spent aronia organic material from Aronia Original Naturprodukte GmbH (Dresden, Germany). Hop production residue was provided by Hopsteiner HHV Hallertauer Hopfenveredlungsgesellschaft m.b.H. (Mainburg, Germany). The soil or soil compost mixtures were filled into aluminum foil trays (33 × 10 cm) and wetted with tap water if needed. Three grams of seeds (= about 1,100 seeds) was sown per replicate. The total aerial tissue of the sprouts with fully expanded cotyledons were harvested after 12 days above the surface, ~2–3 cm long) and immediately frozen in liquid nitrogen and stored at −80°C until lyophylisation. Prior analysis the samples were ground to a homogeneous, fine powder with a Retsch ball mill. All experiments were performed in triplicate.

**Table 1:** Composition of soil and soil compost mixtures.

	**Soil**	**Soil compost mixtures**					
		**Coffee**		**Hop**		**Aronia**	
		**5%**	**10%**	**5%**	**10%**	**5%**	**10%**
pH-Wert in CaCl_2_	5.7	5.7	5.7	6.3	6.5	5.9	5.9
EC μs/cm	1666	1532	1385	2390	2615	1018	981
**[mg/100 g]**							
Mg in CaCl_2_	49	50	46	57	57	44	43
Mg in DL^*^	89	94	88	131	141	87	87
K	148	127	133	498	668	193	229
P	91	75	71	131	141	76	57
Salt content	463	430	387	658	740	279	258
${\text{NO}}_{3}^{-}$	91	75	71	92	104	76	57
${\text{NH}}_{4}^{+}$	<0.2	0.4	0.7	3.7	7.4	0.3	0.6
**[%]**							
C	29.4	32.6	33.0	34.2	33.9	32.6	33.0
N	0.80	0.86	0.90	1.27	1.45	0.82	0.84
C/N	37	38	37	27	23	38	37
S	0.23	0.24	0.20	0.27	0.24	0.24	0.17

* *double lactate extract*.

### Non-targeted analysis of non-volatile metabolites

To assess differences in the composition of metabolites of sprouts grown on pure soil and compost soil mixtures non-volatile metabolites were analyzed by UHPLC-ToF-MS according to Errard et al. (2015, 2016). Ten milligrams of finely powdered tissue was extracted five times with 1.5 mL of 70% methanol acidified with 0.1% formic acid by shaking for 5 min. The volume of the combined supernatants was adjusted to a final volume of 10 mL. The extract was filtered through a 0.2 μm PTFE membrane prior to UPLC-ToF-MS (Agilent Technologies 6230 TOF LC/MS) analysis. Samples (5 μL) were injected into an Agilent Zorbax Extend—C18 Rapid resolution HT (50 × 2.1 mm, 1, 1.8 μm) column. Sample and column temperatures were maintained at 4° and 30°C, respectively. The metabolites were eluted at a flow rate of 0.4 ml min^−1^ using a chromatographic gradient [A: 0–3 min 98% (isocratic), 3–15 min 98–15%, 18 min 15–0%)] of the two mobile phases A; 0.01% aqueous formic acid and B; 0.01% formic acid in acetonitrile. The capillary voltage was set to 3.5 kV in the electrospray source. The source temperature was set to 320°C. The nebulizer gas flow was set to 8 L min^−1^ at 35 psi. Spectra were collected in negative or positive ionization mode at over the *m/z* range 70–1,200.

### Analysis of carotenoids

The carotenoids were extracted three times from 5 mg of freeze dried material using 0.5 ml methanol: tetrahydrofuran solution (1:1, v/v) according to Errard et al. (2015) and Mageney et al. (2016). The extracts were shaken at 1,000 rpm for 5 min at room temperature and centrifuged at 4,500 rpm for 5 min at 20°C. The combined extracts were evaporated in a stream of nitrogen. The extracts were dissolved in 0.02 ml dichloromethane and 0.18 ml of isopropyl alcohol. Prior analysis the solutions were filtered through a 0.2 μM PTFE membrane and kept at 4°C in the auto sampler during the analysis. The separation was performed on a C30-column (YMC Co. Ltd., Japan, YMC C30, 100 × 2.1 mm, 3 μm) on an Agilent Technologies 1290 Infinity UHPLC. Mixtures of methanol, methyl-tert-butyl-ether and water in different volume ratios (solvent A: 81/15/4 and solvent B: 6/90/4) were used as mobile phases at a flow rate of 0.2 ml min^−1^. To enhance the ionization 20 mM ammonium acetate was added to the mobile phases. The pigments were analyzed on an Agilent Technologies 6230 TOF LC/MS equipped with an APCI ion source in positive ionization mode. The gas flow rate was set to 8 L min^−1^ and the temperature to 325°C, the vaporizer temperature to 350°C and the nebulizer pressure was set to 35 psi. The voltage was set to 3,500 V and a fragmentor voltage of 175 V was applied at a corona current of 6.5 μA. Stock solutions of the authentic standards were prepared individually and the concentrations were determined spectro-photometrically using the substance specific wavelengths and extinction coefficients. Identification was achieved by co-chromatography with references substances. External standard calibration curves were used for quantification by dose–response curves.

### Analysis of glucosinolates

Desulfo-glucosinolates were analyzed after a method reported by Witzel et al. (2015). Twenty milligrams of lyophilized sample material were extracted by shaking with 750 μL 70% hot methanol (70°C) for 10 min at 1,400 rpm. As internal standard 100 μL 2-propenyl glucosinolate (sinigrin) (1 mM) were added to the extraction mixture. The samples are centrifuged at 4,500 rpm at room temperature and the supernatant transferred to sample tube. The samples are re-extracted twice with 500 μl of hot methanol. The combined supernatants were applied to an acetic acid-activated DEAE Sephadex A-25 (0.5 mL bed volume) and desulfolated by 75 μL ß-glucuronidase/arylsulfatase over night at room temperature. Desulfo-glucosinolates were obtained by elution with 0.5 mL ultrapure water (2x) and the samples filtered through a SpinX (0.22 μMSpinX Costar Cellulose acetate, 3 min 3000 rpm) prior analysis by UHPLC-DAD (Agilent Technologies 1290 Infinity). The separation was performed on a Poroshell 120 EC-C18 (100 × 2.1 mm, 2.7 μm) column in gradient mode using water and 40% acetonitrile as mobile phases at a flow rate of 0.4 mL min^−1^. The glucosinolate concentrations were calculated using 2-propenyl glucosinolate as an internal standard at a detection wavelength of 229 nm.

### Analysis of phenolic compounds

The analyses were performed based on a modified method by Schmidt et al. (2010). Twenty milligrams of lyophilized sample material were extracted with 600 μL 60% methanol by ultra-sonication followed by 20 min shaking at 1,400 rpm. The sample was pelleted at 12,000 rpm at 20°C and re-extracted by 400 and 200 μL. The combined supernatant was evaporated and re-dissolved in 500 μL of 20% methanol. Prior analysis the samples were filtered through a SpinX (0.22 μM SpinX Costar Cellulose Acetate, 3 min 3,000 rpm). The phenolics were analyzed on an Agilent Technologies 6230 TOF LC/MS equipped with an ESI ion source in positive ionization mode. The gas temperature was set to 350°C at a flow rate of 10 L min^−1^, the vaporizer to 320°C and the nebulizer pressure was set to 35 psi. The voltage was set to 3,500 V and a fragmentor voltage of 350 V was applied. The separation was performed on a Prodigy ODS1 100 A (150 × 2.1 mm, 5 μm) column in gradient mode using 0.5% acetic acid and acetonitrile as mobile phases at a flow rate of 0.4 mL min^−1^. Stock solutions of the authentic standards were prepared individually and identification was achieved by co-chromatography with references substances. External standard calibration curves were used for quantification by dose–response curves.

### Analysis of selected soil properties

The analyses have been performed by validated analytical methods of LUFA (Hoffmann, 1991). The analyses of the soil-pH (A 5.1.1), the EC value, salt content (A 10.1.1) as well as ${\text{NO}}_{3}^{-}$ and ${\text{NH}}_{4}^{+}$ (A 6.1.4.1) have been described by Seck-Mbengue et al. (2017). The total C, N and S concentrations were determined by Dumas combustion using the Elementar Analyser Vario EL (Elementar, Elementar Analysensysteme GmbH, Langenselbold, Germany) according to LUFA A 2.2.5. Minerals have been determined after LUFA (A 6.2.4.1/A 6.2.4.2) using an ICP-OES iCAP 7400 instrument (Thermo Scientific GmbH, Dreieich, Germany) at the following detection wavelengths: P 213.618 nm, K 766.490 nm, and Mg 285.213 nm.

### Data pre-treatment and data analysis

The raw data from the UPLC-ToF-MS analysis were transformed and processed by Mass Profiler Professional (MPP; Version 12.1). The raw data were extracted by using the molecular feature extraction of Mass Hunter B.06.00 using the following settings: small molecules, min. 500 counts, ion species [M+H]^+^, [M-H]^−^, [M+Na]^+^, [M+NH_4_]^+^, H_2_O as neutral loss, peak spacing tolerance 0.025 ppm and a quality score based on mass accuracy, isotope abundance, and isotope spacing of 80%. Raw data files were imported to MPP for recursive workflow. Statistical analysis was carried out by MPP and included one-way-ANOVA followed by *post-hoc* Tukey's HSD test (*p* ≤ 0.05). Compounds subjected to a minimum fold change of 2 were considered for identification. Afterwards, formulas were generated using the above mentioned ions and using a match tolerance of 10 ppm. Putative identification was obtained by using the Metlin database (Vers. 4.0, 24768 compounds). The statistical analysis of the results obtained from the targeted analysis of carotenoids, glucosinolates, and phenolic compounds was performed by Statistica 12. Shapiro–Wilk test has been used to test normal distribution. The means were compared by one-way-ANOVA followed by *post-hoc* Tukey's HSD test (*p* ≤ 0.05).

## Results

### Characteristics of soil and soil compost mixtures

Changes induced by addition of compost to the soil have been investigated and are summarized in Table 1. The pH of the soil compost mixtures was not affected by the addition of coffee, but increased from 5.7 to 5.9 using aronia and to 6.5 using hop compost. The EC value increased after addition of hop compost and was lower using aronia compost and coffee compost. Major changes have been detected after addition of hop compost. Mg, K, P, ${\text{NO}}_{3}^{-}$, and ${\text{NH}}_{4}^{+}$ increased in the hop compost soil mixtures as well as the total salt content. In aronia compost soil mixtures the total salt content was much lower compared to the common soil, however cannot be explained by the changes in Mg, K, P, or ${\text{NO}}_{3}^{-}$ and ${\text{NH}}_{4}^{+}$ and further analyses are required to explain the changed salt contents. The total N was the highest in the hop compost soil mixtures which also yield in a changed C/N ratio compared to the other soils. The carbon contents were in general higher after addition of biological waste as compost, whereas the impact on the total S content was minimal.

### Effects of composts on metabolite profiles

**Figure 1:**
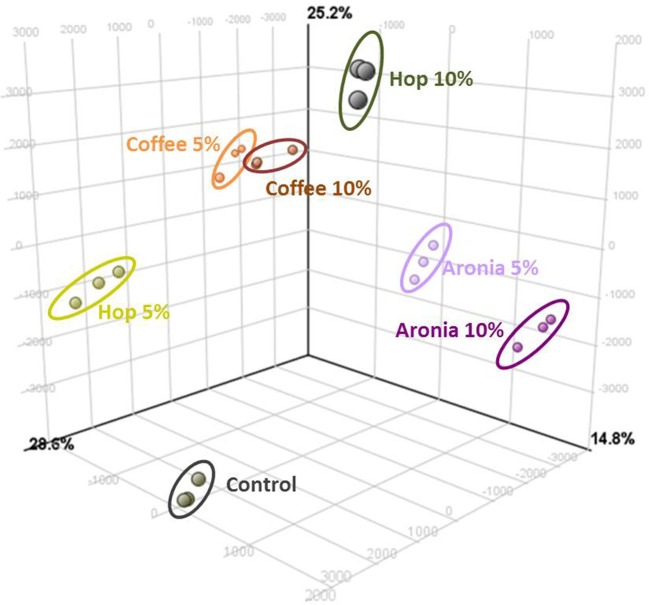
Results of the principal component analysis (PCA) of significant differently abundant compounds (ANOVA *p* ≤ 0.05%, *post-hoc* Tukey HDS, min fold change 2) analyzed by UHPLC-ToF-MS^+/−^.

LC-ToF-metabolomics was used to investigate the effect of biological waste as compost on non-volatile metabolites in pak choi. Therefore, metabolomic differences were combined with statistical analysis to determine the degree of metabolic differences caused by changing soil compositions. To evaluate the effect of organic residues on the metabolome, visualization of the altered metabolite profiles was done using principal component analysis (Figure 1). The first three principal components accounted for 28.6, 25.2, and 14.8% of the variance in pak choi samples, respectively. The samples could be clearly classified by soil conditions. We observed clusters for sprouts grown solely on soil and those grown on different soil-compost mixtures. There is a slightly different response in the sprouts depending on the amount of biological waste inside the soil. The overall composition was reflected by metabolic differences in the primary and secondary metabolite metabolism. We observed changes in the amino acid, energy, hormone, phenylpropanoid, and sugar metabolism (Table 2). For instance the amino acids isoleucine, leucine, methionine, and proline were all increased in sprouts grown on the different composts compared to the control group. Changes in other metabolites of the amino acid metabolism were related to the type and concentration of compost, e.g., threonine increased in sprouts grown on coffee or hop compost or 2-oxoglutarate and oxaloacetate in sprouts grown on coffee or aronia compost. Pyruvate was detected in higher concentration in sprouts grown on coffee compost and 4-hydroxyphenylpyruvate in those grown on hop compost. Glycerinaldehyde-3-phosphate, a metabolite of the sugar metabolism, decreased in any sample grown on compost, whereas galactose, galactose-1-phosphate, and glucose were found in higher concentrations in sprouts grown on coffee compost. Two metabolites of the energy metabolism have been tentatively identified. AMP strongly increased in sprouts grown on coffee or aronia compost, whereas dAMP decreased in samples grown on hop or aronia compost. Different pathways of the hormone metabolism were involved in the response to the usage of composts, such as salicylate, abscisic acid, and jasmonic acid. Salicylate and an epi-jasmonic acid derivative were reduced compared to the control, except for the sprouts grown on 10% coffee compost and cis-abscisate strongly induced by coffee and hop compost. An induction of the ascorbate levels have been found in samples grown on coffee or hop compost and for dehydroascorbate in those grown on coffee or aronia compost. Malate was detected in higher concentrations in sprouts grown on coffee or hop compost compared to the control group. Compounds of the phenylpropanoid metabolism were reduced compared to sprouts solely grown on soil, except for a tentatively identified isorhamnetin derivative which was accumulated in sprouts grown on coffee or hop compost.

**Table 2:** Metabolites affected significantly by organic composts (coffee, hop, aronia) in soil (n.d. not detected; – reduced compared to the control; + increased compared to the control; level of change: weak (+)/(−) (0–1), moderate +/− (1–5), strong ++ (5–20), very strong + + + >100).

	**Coffee**		**Hop**		**Aronia**	
	**5%**	**10%**	**5%**	**10%**	**5%**	**10%**
**AMINO ACIDS METABOLISM**						
L-Isoleucine	+	+	+	+	+	+
Leucine	+	+	+	+	+	+
Methionine	+	+	+	+	(+)	+
Proline	+	+	+	++	+	+
Threonine	+	+	(+)	+	−	−
Tyrosine	−	−	++	+	−	−
Phenylalanine	−	−	−	+		(+)
4-Hydroxy-phenylpyruvate	−	−	++	+	−	−
2-Oxo-3-phenylpropanoate	+	+	−	+	+	+
2-Oxoglutarate	(+)	(+)	−	−	+	+
Oxalacetate	++	++	−	−	+++	+++
Pyruvate	+	(+)	−	−	−	−
**ENERGY METABOLISM**						
AMP	+++	+++	−	−	+++	+++
dADP			−	−	−	−
**HORMONE METABOLISM**						
Salicylate	−	−	−	−	−	−
Salicin	(+)	(+)	−	−	+	+
Benzoate	−	(+)	++	++	++	++
*cis*- Abscisate	+++	+++	+++	+++	−	−
*epi*-Jasmonic acid derivative	−	+	−	−	−	−
**SUGAR METABOLISM**						
Glycerinaldehyde-3-phosphate	−	−	−	−	−	−
Galactose	+	+	−	−	n.d	+
Galactose-1-phosphate	+++	+++	n.d.	n.d.	n.d.	
Glucose	+	+	−	−	n.d.	+
**PHENYLPROPANOID METABOLISM**						
Chrosmic acid	−	−	n.d.	n.d.		−
Galic acid	−	(−)		−	−	−
Chlorogenic acid derivative	−	−	(−)	−	−	−
Isorhamnetin derivative	+	+		+	−	−
**OTHERS**						
Ascorbate	+	+	+	+		−
Dehydroascorbate	(+)	(+)	−	−	+	+
Malate	+	+	+	+	−	−
Isocitrate	+	+	−	−	+	+
Citrate	+	+	−	−	+	+
Fumarate	+	(+)	−	−	−	−

### Effect of compost on carotenoids, glucosinolates, and phenolic compounds

On the basis of the putatively identified compounds we expected changes in valuable secondary plant metabolites and carried out a targeted analysis of carotenoids, glucosinolates, and phenolic compounds in the lyophilized sample material.

**Figure 2:**
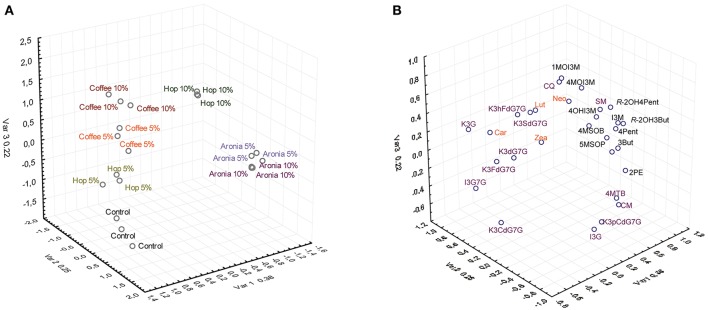
Results of the factor analysis of the targeted analysis of glucosinolates, phenolic compounds, and carotenoids by treatment **(A)** and compound **(B)**. Abbreviations see Table 3.

The factorial analysis of major carotenes, xanthophylls, glucosinolates, and phenolic compounds revealed that the different soil mixtures had noticeable impacts on the secondary plant metabolite profile (Figure 2).

The control group (sprouts grown on pure soil) and sprouts grown on soil mixtures with coffee, hop, or aronia composts show altered secondary plant metabolite profiles. Whereas slight differences were observed for the profiles of sprouts grown on coffee compost (5 and 10%) or aronia compost (5 and 10%), we observed higher variations among sprouts grown on hop compost (5 and 10%) (Figure 2A). Carotenoids contribute importantly to changes in sprouts grown on coffee and hop compost (5%), and glucosinolates in samples grown on aronia (5 and 10%) and hop compost (10%). The phenolic compounds show a more structure-specific response and contribute to the different metabolite profiles in sprouts grown on hop, aronia, or coffee compost (Figure 2B). More in detail, we estimated single carotenoid, glucosinolate, and phenolic compounds variability in sprouts of pak choi grown on pure soil (control) and compost-soil mixtures (Table 3).

**Table 3:** Carotenoids, glucosinolates, and phenolic compounds in pak choi grown on soil and soil-organic compost mixtures.

	**Control**	**Coffee**		**Hop**		**Aronia**	
		**5%**	**10%**	**5%**	**10%**	**5%**	**10%**
**CAROTENOIDS**							
Car	27.4 ± 2.1a	53.1 ± 16.5ab	79.1 ± 23.5ab	**34.0 ± 1.7b**	**35.3 ± 1.8b**	**31.2 ± 2.4b**	60.2 ± 3.0ab
Lut	236.9 ± 46.6a	**753.9 ± 26.3b**	**703.9 ± 24.5cd**	**619.9 ± 31.0c**	**966.9 ± 48.3e**	**493.3 ± 32.5b**	**488.5 ± 24.4b**
Neo	51.6 ± 7.5a	**147.3 ± 4.8b**	**141.3 ± 17.7b**	**132.7 ± 6.6b**	**186.9 ± 9.3c**	59.5 ± 3.2a	55.3 ± 2.8a
Zea	22.4 ± 1.1a	**27.9 ± 1.5b**	21.8 ± 0.3a	**28.9 ± 1.4b**	**37.9 ± 1.9c**	**26.7 ± 0.5b**	20.1 ± 1.0a
**GLUCOSINOLATES**							
4MTB	0.34 ± 0.020e	**0.19 ± 0.001c**	**0.12 ± 0.049b**	**0.28 ± 0.011d**	**0.06 ± 0.004a**	**0.11 ± 0.010ab**	**0.19 ± 0.001c**
4MSOB	0.26 ± 0.019bc	0.32 ± 0.026c	0.27 ± 0.051bc	0.28 ± 0.060bc	**0.08 ± 0.008a**	0.19 ± 0.036b	0.20 ± 0.001b
5MSOP	0.55 ± 0.014c	0.56 ± 0.017c	0.52 ± 0.013c	**0.66 ± 0.077d**	**0.19 ± 0.003a**	**0.39 ± 0.031b**	**0.36 ± 0.004b**
3But	3.31 ± 0.096d	**2.71 ± 0.060c**	**2.53 ± 0.069c**	3.49 ± 0.162d	**0.76 ± 0.006a**	**1.71 ± 0.034b**	**1.51 ± 0.002b**
4Pent	2.95 ± 0.025f	**2.70 ± 0.069e**	**2.51 ± 0.082d**	2.86 ± 0.114ef	**0.53 ± 0.002a**	**1.27 ± 0.024c**	**0.96 ± 0.003b**
(*R*)-2OH3But	4.07 ± 0.186d	**3.59 ± 0.076c**	**3.50 ± 0.124c**	4.20 ± 0.203d	**0.81 ± 0.014a**	**1.19 ± 0.023b**	**0.6 ± 0.001a**
(*R*)-2OH4Pent	0.74 ± 0.030cd	0.71 ± 0.014cd	0.80 ± 0.078d	0.67 ± 0.022c	**0.13 ± 0.003a**	**0.25 ± 0.008b**	**0.11 ± 0.001a**
I3M	0.29 ± 0.073b	0.23 ± 0.006b	0.25 ± 0.038b	0.27 ± 0.018b	**0.07 ± 0.004a**	**0.11 ± 0.005a**	**0.06 ± 0.000a**
4OHI3M	0.31 ± 0.053bc	0.33 ± 0.031c	0.33 ± 0.120c	0.32 ± 0.059c	**0.09 ± 0.004a**	0.20 ± 0.044abc	0.16 ± 0.004ab
4MOI3M	0.14 ± 0.003d	**0.30 ± 0.014f**	**0.22 ± 0.030e**	**0.11 ± 0.009c**	**0.04 ± 0.001ab**	**0.06 ± 0.006b**	**0.02 ± 0.000a**
1MOI3M	0.16 ± 0.068b	0.22 ± 0.013b	**0.74 ± 0.040c**	0.16 ± 0.008b	**0.05 ± 0.002a**	**0.05 ± 0.002a**	**0.01 ± 0.000a**
2PE	0.43 ± 0.01f	**0.27 ± 0.01d**	**0.22 ± 0.03c**	**0.36 ± 0.01e**	**0.09 ± 0.00a**	**0.14 ± 0.01b**	**0.17 ± 0.00b**
Aliphatic GS	e	**d**	**d**	e	**a**	**c**	**b**
Indolic GS	b	b	**c**	b	**a**	**a**	**a**
Aromatic GS	f	**d**	**c**	**e**	**a**	**b**	**b**
**PHENOLIC COMPOUNDS**							
CM	28.39 ± 0.74e	**0.34 ± 0.07ab**	**0.28 ± 0.13a**	**16.36 ± 1.38d**	**13.85 ± 1.17c**	**2.58 ± 1.35b**	**0.06 ± 0.00a**
CQ	0.6 ± 0.06bc	0.57 ± 0.05b	**2.22 ± 0.11d**	0.59 ± 0.02b	**0.75 ± 0.03c**	**0.34 ± 0.01a**	**0.22 ± 0.01a**
SM	1105.67 ± 56.80cd	1117.34 ± 60.01d	984.15 ± 32.83c	**790.69 ± 33.62b**	**778.64 ± 19.96b**	**721.54 ± 6.64b**	**574.02 ± 3.99a**
I3G	1.22 ± 0.67b	**0.46 ± 0.01a**	**0.45 ± 0.06a**	2.07 ± 0.26b	1.39 ± 0.20b	**0.63 ± 0.01a**	1.52 ± 0.00b
I3G7G	0.82 ± 0.02a	**5.23 ± 0.49b**	**7.63 ± 0.25c**	0.87 ± 0.22a	0.73 ± 0.03a	**28.53 ± 1.91e**	**16.16 ± 0.33d**
K3G	2.10 ± 0.35a	4.97 ± 0.16a	**7.69 ± 0.05d**	3.59 ± 0.17a	**5.95 ± 0.11ab**	**8.32 ± 0.44d**	**6.07 ± 0.07c**
K3CdG7G	18.23 ± 1.48d	**8.41 ± 0.28a**	**7.83 ± 0.58a**	**15.05 ± 0.63c**	**9.38 ± 0.44ab**	**11.87 ± 2.54bc**	**9.69 ± 0.23ab**
K3dG7G	95.69 ± 4.28cd	91.52 ± 6.13c	102.59 ± 3.66d	**58.20 ± 1.99b**	**42.78 ± 1.48a**	**115.54 ± 2.21e**	84.45 ± 0.50c
K3FdG7G	130.28 ± 9.17b	161.17 ± 11.40bc	165.25 ± 7.12c	**105.08 ± 10.70a**	**80.81 ± 7.74a**	**191.13 ± 9.03d**	**164.87 ± 0.51c**
K3SdG7G	593.74 ± 31.83c	**748.00 ± 41.73ef**	**787.18 ± 21.89f**	**468.28 ± 19.73b**	**392.74 ± 2.85a**	**664.71 ± 0.60de**	619.07 ± 4.43cd
K3hFdG7G	151.28 ± 6.40c	168.23 ± 9.72c	**188.85 ± 7.42d**	**129.52 ± 6.17ab**	**131.01 ± 4.09b**	**183.58 ± 2.93d**	**108.85 ± 0.71a**
K3pCdG7G	113.03 ± 10.47b	**101.00 ± 6.92a**	**101.70 ± 5.08a**	**97.64 ± 9.48a**	**90.59 ± 3.74a**	160.92 ± 0.62b	**134.27 ± 2.29c**

*Means [μg/g DW] were compared by one-way-ANOVA followed by post-hoc Tukey's HSD test (p ≤ 0.05). Letters indicate significant differences within a group (in alphabetical order from the lowest to highest) and bold numbers show significant difference compared to the control group (post-hoc Dunnet's test, p ≤ 0.05). Each value represents the mean of three samples ± S_E_*.

*Carotene: Car, β-Carotene; Xanthophylls: Lut, Lutein; Neo, neoxanthin; Zea, Zeaxanthin; aliphatic Glucosinolates: (GS): 4MTB, 4-Methylthio-butyl GS; 4MSOB, 4-Methyltsulfinly-butyl GS; 5MSOP, 5-Methylsufinyl-pentyl GS; 3But, 3-Butenyl GS; 4Pent, 4-Pentenyl GS, (R)-2OH3But, (R)-2-Hydroxy-3-butenyl GS; (S)-2OH3But, (S)-2-Hydroxy-3-butenyl GS; 2OH4Pent, 2-Hydroxy-4-pentenyl GS; indolic GS: I3M, Indol-3-ylmethyl GS; 4OHI3M, 4-Hydroxyindol-3-ylmethyl GS; 4MOI3M, 4-Methoxyindol-3-ylmethyl GS; 1MOI3M, 1-Methoxyindol-3-ylmethyl GS; aromatic GS: 2PE, 2-Phenylethyl GS; Hydroxybenzoeic acids: CM, Caffeoylmalate; CQ, Caffeoylquinicacid; SM, Sinapoylmalate; Flavonoids: I3G, Isorhamnetin-3-O-glucoside; I3G7G, Isorhamnetin-3-O-glycoside-7-O-glycoside; K3G, Kaempferol-3-O-glucoside; K3CdG7G, Kaempferol-3-O-caffeoyl-diglucoside-7-O-glucoside; K3dG7G, Kaempferol-3-O-diglucoside-7-O-glucoside; K3FdG7G, Kaempferol-3-O-feruloyl-diglucoside-7-O-glucoside; K3SdG7G, Kaempferol-3-O-sinapoyl-diglucoside-7-O-glucoside; K3hFdG7G, Kaempferol-3-O-hydroxyferuloyl-diglucoside-7-O-glucoside; K3pCdG7G, Kaempferol-3-O-p-coumaroyl-diglucoside-7-O-glucoside*.

#### Effect of compost on carotenoids

Highest changes were observed for the xanthophyll lutein (up to 3.2-fold), which is also the major carotenoid in pak choi sprouts. The concentration in the control group was 236 ± 46.6 μg/g and with all treatments showing a significant increase and the largest increase found in sprouts grown on 10% hop compost. The lutein concentration in this sample group was 966.9 ± 48.3 μg/g. In the treatments, the concentration of neoxanthin was found to increase by up to 3-fold. Major changes compared to the control group (51.6 ± 7.5 μg/g) were detected again for sprouts grown on 10% hop compost (186.9 ± 9.3 μg/g). The changes in the xanthophyll zeaxanthin were less pronounced with max 1.7-fold change in case of sprouts grown on 10% hop compost (22.4 ± 1.1 μg/g control compared to 37.9 ± 1.9 μg/g 10% hop compost), too. The alterations in the β-carotene contents were minor compared to the variations observed for the xanthophylls. Slightly higher concentrations have been detected in all sprouts grown on compost, but significant effects were only found in the samples grown on hop compost or 5% aronia compost.

#### Effect of compost on glucosinolates

The total amount of the glucosinolates decreased in all sprouts except in the sample where 5% of the soil was replaced by compost derived from hop residue. In case of compost from coffee the reduction was from 13.62 ± 0.014 μg/g in the control to 12.19 ± 0.153 μg/g and 12.17 ± 0.118 μg/g in sprouts grown on 5 and 10% coffee compost, respectively. Higher losses have been found after addition of aronia (5.66 ± 0.105 μg/g 5% aronia and 4.36 ± 0.079 μg/g 10% aronia) or hop compost (2.94 ± 0.006 μg/g 10% hop) (Table 3). In all samples grown on soil-compost mixtures the aliphatic glucosinolates were reduced. Highest losses were found in those samples grown on aronia compost (5 and 10%) and on hop compost (10%). Using aronia compost the concentrations dropped by 0.59 (5% aronia compost) and 0.68 (10% aronia compost), from 12.29 ± 0.11 μg/g to 5.10 ± 0.11 μg/g and 3.94 ± 0.04 μg/g. In the pak choi sprouts grown on 10% hop compost the loss of aliphatic glucosinolates was 0.79 corresponding to 2.5 ± 0.01 μg/g. Interestingly, in sprouts grown on coffee compost, just the concentration of the aliphatic 4-methylthiobutyl glucosinolate was significantly reduced. The indole glucosinolates were significantly affected using coffee (5 and 10%), aronia (5 and 10%) or hop compost (10%). Noteworthy, in sprouts grown on the coffee compost the concentration of the methoxylated indole glucosinolates increased significantly. The 4-methoxyindole-3-ylmethyl glucosinolate was significantly affected by compost containing 5% coffee (0.30 ± 0.014 μg/g) residues compared to the control (0.14 ± 0.003 μg/g), whereas the 10% coffee compost affected more the 1-methoxyindole-3-ylmethyl glucosinolate (0.16 ± 0.068 – 0.74 ± 0.040 μg/g). The precursor of both, the indole-3-ylmethyl glucosinolates, kept almost unaffected compared to the concentration in sprouts grown on pure soil. In contrast, soils mixed with aronia (5 and 10%) and hop (10%) reduced significantly the concentration of all indole glucosinolates compared to the control. The aromatic 2-phenylethyl glucosinolate was significantly reduced in all samples grown on soil-compost mixtures compared to pure soil (Table 3).

#### Effect of compost on phenolic compounds

The concentration in the phenolic compounds (2154.43 ± 93.70 μg/g) did not change (5 and 10% coffee compost, and 5% aronia compost) or were lower (5 and 10% hop compost, and 10% aronia compost compared to pure soil). In pak choi sprouts grown on 5% hop compost the concentration was reduced by 0.25 (1665.94 ± 76.36 μg/g), on 10% hop compost by 0.31 (1525.24 ± 33.01 μg/g, and on 10% aronia compost by 0.23 (1719.27 ± 7.23 μg/g).

More specifically, single phenolic compounds showed a particular response in respect of the various soil composts (Table 3); e.g., caffeoylmalate and kaempferol-3-*O*-caffeoyl-diglucoside-7-*O*-glucoside were reduced from control values of 28.39 ± 0.74 μg/g by up 100-fold and 18.23 ± 1.48 μg/g by 2-fold, respectively. The concentrations of isorhamnetin-3-*O*-glycoside-7-*O*-glycoside and kaempferol-3-*O*-sinapoyl-diglucoside-7-*O*-glucoside were higher in samples grown on coffee compost (7.63 ± 0.25 μg/g and 748.00 ± 41.73, respectively) and aronia compost (16.16 ± 0.33 μg/g and 664.7 ± 0.60 μg/g, respectively). To summarize, the addition of hop compost (5 and 10%) to the soil resulted in a decrease of malates and all kaempferol glycosides except for kaempferol-3-*O*-glucoside, whereas the use of aronia compost (5 and 10%) showed a decrease of malates but an increase of most kaempferol glycosides depending on the compost percentage added to the soil. However, the coffee composts (5 and 10%) were presented by decrease of caffeoylmalate to a minimum of 0.28 ± 0.13 μg/g, but only slight or no changes in the kampferol glycosides showing an increase of kaempferol-3-*O*-sinapoyl-diglucoside-7-*O*-glucoside to 787.18 ± 21.89 μg/g, kaempferol-3-*O*-glucoside to 7.83 ± 0.58 μg/g, and kaempferol-3-*O*-hydroxyferuloyl-diglucoside-7-*O*-glucoside to 188.85 ± 7.45 μg/g.

## Discussion

### Effects of composts on metabolite profiles

In this study profiling methods and targeted analyses have been used to explore the overall impact of biological waste of food chain production used as composts on the metabolite profile of pak choi sprouts. Metabolomic approaches for studying changes induced by biotic (Errard et al., 2015) and abiotic factors (Jorge et al., 2016) became an important analytical tool in plant science (Witzel et al., 2015). The metabolite profile differed when comparing sprouts grown composts and control plants grown solely on soil. The overall composition was reflected by metabolic differences in the primary and secondary metabolism.

Interestingly, changes in diverse hormone signaling pathways have been observed. It is well-known that abscisic acid (Taylor et al., 2000), salicylic acid (Janda et al., 2007), and jasmonic acid (Creelman and Mullet, 1995) act as signals in plants to response to biotic and abiotic challenges alone or in combination. These changes lead to alternation in primary and secondary metabolites of which the latter one will be discussed specifically in the following subchapters. Whereas, many amino acids were reduced in response to biotic stress (Errard et al., 2016), in the present study the application of composts increased their concentrations. This accounts also for some sugars. The changes in ascorbate might be also correlated with changes in the xanthophyll cycle, where one of the key enzymes, the violaxanthin de-epoxidase, requires ascorbate as co-substrate and the enzymatic activity is pH-depended (Arnoux et al., 2009). The glucosinolate levels also depend on ascorbate, because ascorbate influences the myrosinase activity—the enzyme responsible for the hydrolyzation of glucosinolates to the bioactive breakdown products (Hasapis and Macleod, 1982). But, in our experimental setup the myrosinase and glucosinolates kept separated from each other and further research is needed to evaluate the possible interaction between ascorbate and glucosinolate concentrations.

Responses to abiotic stress are dynamic and complex and to get a comprehensive understanding further systematic studies are required to understand the specific changes induced by the different composts.

### Effect of compost on carotenoids, glucosinolates, and phenolic compounds

We focused our recent study on the secondary metabolites because they are associated with health beneficial properties. Carotenoids are well-known antioxidants and they can lower the risk of chronic diseases, including cardiovascular diseases (Gammone et al., 2017) and type-2 diabetes mellitus (Akbaraly et al., 2008), and several types of cancer (Bolhassani, 2015). Lutein and zeaxanthin can prevent age-related eye diseases such as macular degeneration (Mares, 2016; Frede et al., 2017). Crops have been bred or engineered to increase β-carotene as precursor of vitamin A (Bai et al., 2011). Glucosinolates, as characteristically secondary metabolites in *Brassica* vegetables, are of interest due to the wide range of health-promoting properties of their breakdown products, e.g., for anti-carcinogenic (Traka and Mithen, 2009; Wu et al., 2012), anti-diabetic (Waterman et al., 2015; Guzman-Perez et al., 2016), and anti-inflammatory effects (Bentley-Hewitt et al., 2014; Herz et al., 2016). For human health phenolic compounds are of special interest due to their antioxidant activity (Zietz et al., 2010), as well as anti-inflammatory and anti-cancerogenic effects (Pan et al., 2010; Chen and Chen, 2013).

Structure-specific responses of carotenoids, glucosinolates, and flavonoid glycosides, in *Brassica* species are also found in response to other eco-physiological conditions such as light or temperature (Schonhof et al., 2007; Neugart et al., 2012; Mageney et al., 2016). However, the sprouts were grown under the same environmental conditions and therefore interacting climatic effects are not expected.

#### Effect of compost on carotenoids

The structure-specific response of phenolic compounds as well as the variation in carotenoids and glucosinolates might be partly due to the various nitrogen contents of these composts. We measured the total nitrogen in the organic composts. The highest total nitrogen content was found in the hop compost and slightly higher values in the coffee and aronia compost soil mixtures. Plants response differently to changes of nitrogen concentration and form in soil. For instance, reduction in nitrogen fertilization yield in a reduction of carotenoids in lettuce (Becker et al., 2015), but carotenoid pigments increased in response to increasing nitrogen concentrations in kale (Kopsell et al., 2007) as they did in this study. In other studies, the carotenoid concentrations were not affected such as in leafy *Brassica* species (e.g., Fallovo et al., 2011). Under organic farming practices, higher concentrations of carotenoids were detected in cauliflower (Lo Scalzo et al., 2013). The content of carotenoids in carrot roots and human diets was not significantly affected by the agricultural production system (organic vs. conventional) or year, despite differences in fertilization strategy and levels (Soltoft et al., 2011), whereas β-carotene was found to be higher in organically grown *Brassica* species (Kapusta-Duch et al., 2014). This is in accordance to this study, where higher carotenoid concentrations were detected in sprouts grown on compost.

Carotenoids also react in response to stresses. In our study, neoxanthin increased in sprouts grown on soil containing composts from coffee and hop. Neoxanthin positively influences ABA accumulation in response to dehydration (North et al., 2007). However, ABA is only one plant hormone influenced by growing conditions and the metabolic profiling revealed that changes not only occurred in the abscisic acid signaling, but also in the salicylic acid, and jasmonate pathways (Table 2). Salicylic acid concentrations have been positively associated with carotenoid concentrations in maize and Indian mustard (Khodary, 2004; Thakur and Sohal, 2014), whereas methyl jasmonate either reduced the degradation of carotenoids in post-harvest lettuce (Kim et al., 2006) or increased β-carotene as well as mannitol in broccoli sprouts (Natella et al., 2016). The exogenous application of ABA, JA, and SA was positively correlated with photosynthetic pigment concentrations in turnip (Thiruvengadam et al., 2016). Therefore, the changes in plant hormones are one possible approach to explain the higher carotenoid concentrations in the present study. An additional factor might be the regulation of the violaxanthin de-epoxidase by ascorbate contributing to the accumulation of xanthophylls (Arnoux et al., 2009).

#### Effect of compost on glucosinolates

Glucosinolates are nitrogen containing compounds suggesting that nitrogen supply strongly affect glucosinolate concentration. The effect of nitrogen fertilization on changes in glucosinolates in kale has been reported recently. Increasing nitrogen concentrations in the soil results in reduction of aliphatic glucosinolates and an activation of the glucosinolate catabolic process was observed under ammonium nutrition (Groenbaek et al., 2014; Marino et al., 2016). However, increasing glucosinolate contents were found in broccoli and red cabbage grown on organic soil (Meyer and Adam, 2008). But, the effect of organic soil seems to be genotype-dependent. Picchi et al. (2012) found that the total glucosinolate content as well content of the major glucosinolates decreased in cauliflower grown on organic fields compared to cauliflower grown under conventional conditions. But, these effects were shown just for one cultivar; the glucosinolates in the cultivar Magnifico showed the opposite effect and were increased.

The factor analysis revealed that the aronia compost in both concentrations and hop compost (10%) had the strongest effect on the glucosinolates (Figure 2). In sprouts grown on these compost mixtures all individual glucosinolates decreased. This compost induced glucosinolate reduction could be due to a unbalanced ratio of nitrogen and sulfur in the soil: an optimal or high nitrogen supply combined with an insufficient sulfur supply leads to a decrease of aliphatic and increase of indole glucosinolates (Li et al., 2007). But, the optimal ratio of N/S is not only species-specific, but structure-specific, too. High N/S ratio promote the formation of aliphatic and indolic glucosinolates in broccoli florets (Schonhof et al., 2007), but in turnips a lower ratio of N/S lead to an increase of aromatic glucosinolates (Li et al., 2007). However, in our experiments the N/S ratio does not explain the decreases or increases of the individual glucosinolates. Furthermore, the ratio of alkenyl to hydroxyalkenyl glucosinolates is changed from about 1.3 to 2.1 (5%) and 3.5 (10%) in sprouts grown on aronia compost. It might be that aronia residues in the soil influence the conversion of alkenyl to hydroxyalkenyl glucosinolates due to the induction of the 2-oxo acid dependent dioxygenase (*BrGSL-OH*) as higher concentrations of 2-oxo acids have been detected in sprouts grown on aronia compost in this study. In contrast, in the coffee compost samples the aliphatic glucosinolates concentrations are slightly decreased or unchanged. Only the methoxylated indole glucosinolates (4-methoxyindole-3-ylmethyl and 1-methoxyindole-3-ylmethyl glucosinolate) increased significantly in pak choi sprouts grown on coffee compost mixtures, which might be due to the ammonium nutrition (Marino et al., 2016). The authors determined different expression levels depending on the nitrogen source: higher expression levels of *CYP79B2/B3* in the indole glucosinolate pathway under nitrate nutrition, and a higher expression of genes involved in the aliphatic glucosinolate pathway under ammonium nutrition. The paradox is that under ammonium nutrition the transcript levels for the genes of the indolic glucosinolate biosynthesis pathway were reduced, but at metabolite level the indolic glucosinolates were increased in *Arabidopsis* and broccoli (Marino et al., 2016). Furthermore, the gene expression of *CYP81F1/F2/F3/F4*, involved in the methoxylation of the indole-3-ylmethyl glucosinolate to the methoxylated indole glucosinolates, might be induced by ammonium. Further investigations are needed to shed light on links between nitrogen and glucosinolate metabolism.

Previous experiments showed a regulation of the glucosinolate metabolism by the salicylic acid or jasmonate pathways. In pak choi sprouts an induction of glucosinolates was observed by methyl jasmonate treatment, but no changes were observed after treatment with methyl salicylate (Wiesner et al., 2013a). Other researchers observed an increase of aliphatic and indole glucosinolates after salicylic acid treatment (Kiddle et al., 1994). The interaction of signaling molecules is more complex and regulated by different positive and negative back loops. Also it could be demonstrated in that a drought-induced accumulation of aliphatic glucosinolates is related to ABA formation *B. juncea* (Tong et al., 2014).

#### Effect of compost on phenolic compounds

The structure-specific response of phenolic compounds might be partly due to the various nitrogen contents of these composts. A negative correlation between specific flavonoid aglycones (kaempferol and quercetin) and nitrogen fertilization in *Brassica juncea* and between flavonoid glycosides and nitrogen fertilization in lettuce has been proven previously (Fallovo et al., 2011; Becker et al., 2015). Also in kale a species-specific response on changes in nitrogen content has been reported for phenolic compounds (Groenbaek et al., 2014). In the kale cv. Reflex the total flavonoid glucosides were significantly reduced, which was in this species genotype mainly related to the changes in quercetin glucosides while kaempferol and isorhamnetin glycosides were less susceptible to higher nitrogen fertilization. However, the main compounds in pak choi and kale are monoacylated kaempferol and quercetin tri- and tetraglycosides which were reduced in kale with higher dose of nitrogen fertilization (Groenbaek et al., 2014). For the pak choi sprouts in this study the monoacylated kaempferol glycosides were reduced with soil-hop compost mixtures assuming a negative correlation of nitrogen and flavonoid glycosides. However, the results were partly different for the three soil compost mixtures tested which leads to the conclusion that nitrogen is not the only influencing factor and the effect of soil coffee and soil aronia compost cannot be explained like this. Organically grown plants are considered to protect themselves better and form phytoalexins like flavonoids (Asami et al., 2003). Asami et al. (2003) found in organically grown plants (marionberry, strawberry and maize) higher total phenolic contents than in conventionally produced plants. In tomatoes quercetin, kaempferol, and narigenin were higher in organically grown plants compared to conventional grown plants in a 10 year trial (Mitchell et al., 2007). Biologically food waste can be considered as organically fertilization and the flavonoid glycosides in pak choi are mainly decreased. There is the need to study other underlying mechanisms that triggers the production of flavonoids. Apart from nitrogen supply, hormones, and other signaling compounds released as roots exudates could contribute to the modulated secondary plant metabolite profiles (Broeckling et al., 2008). Interestingly, we found specific reactions for sprouts grown on soil-aronia composts mixtures, e.g., kaempferol-3-*O*-feruloyl-diglucoside-7-*O*-glucoside increased significantly. It might be speculated that this is the result of higher concentrations in the root environment and lower active release rates by the roots. Further investigations should also include the analysis of modulated soil microorganism communities.

Moreover, jasmonate, salicylate, and abscisic acid are generally known to enhance flavonoids and hydroxycinnamic acids (Chen et al., 2006; Sandhu et al., 2011), but this cannot directly be linked to the present results.

## Conclusion

Interestingly, addition of biological waste of the food production chain as compost had strong effects on plant metabolites profiles in sprouts of pak choi. Different composts of the food production chain impact differently the plant metabolite profile of pak choi sprouts. The usage of coffee, aronia or hop composts incorporated in soil increased the concentration of carotenoids and decreased the amount of glucosinolates and phenolic compounds being associated with health beneficial properties of vegetables. The usage of compost might be besides the application of chemical and physical elicitors another possibility to target the accumulation of specific metabolites e.g., for the production of carotenoids to prevent vitamin A deficiency or age-related macular degenerative diseases. However, taking into account the intrinsic quality and health effects of vegetables that is in large measure driven by the secondary plant metabolites unintentional changes such as observed for glucosinolates and phenolic compounds need further investigations. Further studies are necessary and would shed light on the metabolome dynamics and underlying mechanism responsible for the changes induced by composts.

## Author contributions

SB designed the study in cooperation with MW-R, HR, and KF. MW-R, SN, and SB carried out the plant experiments, contributed to interpretation of data and drafted the manuscript. SB developed the mass spectrometric method for the non-targeted analyses and performed statistics. EJ contributed to the non-targeted analysis. KF analyzed the carotenoids and chlorophylls, SN analyzed the phenolic compounds and MW-R and MS the glucosinolates. TH, HR, and MS critically revised the manuscript. All authors read and approved the final manuscript.

### Conflict of interest statement

The authors declare that the research was conducted in the absence of any commercial or financial relationships that could be construed as a potential conflict of interest.
